# Initial Experience Using the Radiofrequency Needle Visualization on the Electroanatomical Mapping System for Transseptal Puncture

**DOI:** 10.1155/2020/5420909

**Published:** 2020-06-20

**Authors:** Silvia Guarguagli, Ilaria Cazzoli, Aleksander Kempny, Michael A. Gatzoulis, Sabine Ernst

**Affiliations:** ^1^Department of Cardiology, Royal Brompton and Harefield Hospital and Imperial College London, London, UK; ^2^Division of Cardiology, Fondazione IRCCS Policlinico San Matteo, School of Cardiovascular Disease, University of Pavia, Pavia, Italy

## Abstract

**Introduction:**

Transseptal puncture (TSP) is a routine access route in patients with left-sided ablation substrates and is performed safely on fluoroscopy (+/− echocardiographic guidance). We report on our experience using a radiofrequency (RF) needle in an unselected group of patients to demonstrate safety and usefulness of direct tip visualization on the 3D electroanatomical mapping (EAM) system with specific emphasis on total radiation exposure.

**Methods and Results:**

We retrospectively reviewed 42 consecutive left-sided ablation procedures with TSP performed using an RF needle guided by fluoroscopy and/or EAM visualization by a single operator. The procedures included atrial fibrillation (*n* = 33), atrial tachycardia (*n* = 8), and ventricular tachycardia (*n* = 1) ablations. Fourteen of 41 patients had congenital heart disease, including 9 patients with previous septal closure. Twenty-two patients had at least one previous TSP. All TSPs were performed successfully and without complications. The overall median fluoroscopy time amounted to 3.2 min and median exposure of 199.5 *µ*Gy^*∗*^m^2^. In a subgroup of patients (*n* = 27), the RF needle was visualized on the EAM system: median radiation time was 0.88 (interquartile range: 0–3.4) min and median exposure 33.5 [0–324.8] *µ*Gy^*∗*^m^2^.

**Conclusions:**

TSP using an RF needle is an effective technique, also in congenital patients with artificial patch material and in normal patients with multiple previous TSPs. Moreover, the RF needle tip visualization on EAM allows a low (or even zero) fluoroscopy approach.

## 1. Introduction

Transseptal puncture (TSP) is a routine access route in patients with left-sided ablation substrates, traditionally performed using fluoroscopy (+/− echocardiographic guidance). For many operators, this is the main part of the procedure that requires radiation exposure, while mapping and ablation are mostly, if not exclusively, carried out guided by a 3D electroanatomical mapping (EAM) system.

In the attempt to reduce radiation exposure, the use of EAM has been advocated strongly [1]; the effect, however, depends greatly on the operator's experience with some reports showing only a modest reduction of total fluoroscopy exposure despite using the so-called “nonfluoroscopy systems” [2]. However, especially in paediatric electrophysiology (EP), a number of centres have reported on ZERO fluoroscopy exposure procedures [3–5]. Lastly, not only the patient but also the operator and the catheterization laboratory staff benefit from low radiation exposure [6].

We report herewith on our experience of employing a radiofrequency (RF) needle (NRG, Baylis Medical, Toronto, Canada) for TSP in an unselected group of patients undergoing left-sided ablation procedures.

## 2. Methods

### 2.1. Patients

We retrospectively reviewed all consecutive patients who underwent a single or double TSP using an RF needle for a left-sided ablation procedure from January 2015 to July 2017. All procedures were performed by the same operator and all patients were included. Initially, the RF needle was used only when the conventional TSP failed or in presence of an artificial septum. Once the visualization of the RF needle was available on the EAM, we also performed de novo procedures in this fashion for any patient that required TSP.

In addition to baseline characteristics (age, sex, body max index (BMI), renal dysfunction, and previous interventions), procedural data such as total procedural time, total procedural fluoroscopy time (FT, measured in minutes from groin puncture to the end of the procedure), and fluoroscopy exposure (Dose Area Product, surrogate measurement for the entire amount of energy received by the patient, measured in *μ*Gy·m [2]) were recorded directly from the imaging system (AXIOM Artis, Siemens, Germany) in a single catheterization laboratory (Cath lab#4, Royal Brompton and Harefield NHS Foundation Trust). All patients were admitted for overnight observation, had a transthoracic echocardiogram, and were monitored on telemetry after the procedure.

### 2.2. 3D Imaging and 3D Mapping System

All but one patient underwent cardiac imaging prior to the ablation procedure, either by contrast-enhanced computed tomography (CT) scan or by noncontrast cardiac magnetic resonance (CMR) imaging as it is a standard at our institution. For CMR, a free-breathing, diaphragm-navigated, balanced steady-state free precession sequence with 3D reconstruction was performed to image the whole heart [7]. All preacquired 3D imaging data were processed to obtain individual 3D reconstructions of all cardiac chambers and vessels to be fused with the EAM 3D maps (POLARIS software, Biosense Webster, Brussels, Belgium).

The electroanatomical mapping system CARTO3 (Biosense Webster, Brussels, Belgium) was used in all but two procedures, which were performed with the Rhythmia (Boston Scientific, Marlborough, MA) 3D mapping system. For 3 procedures (#19, #28, and #41) in patients with complex congenital heart disease, the remote magnetic navigation system (NiobeII, Stereotaxis Inc., St. Louis, MS) was used in combination with CARTO [8].

### 2.3. Invasive EP Procedure

After obtaining written informed consent, the procedures were performed under deep assisted sedation or general anesthesia in the presence of a cardiac anaesthetist and with continuous invasive arterial blood pressure monitoring. A transoesophageal echocardiography excluded clots in the left atrial appendage (LAA) prior to the procedure. Femoral venous accesses were gained using ultrasound guidance. Subsequently, via the left femoral vein, an octapolar diagnostic catheter (St. Jude Medical, Belgium) or a decapolar catheter (BARD Medical, Covington, Georgia) was positioned across the tricuspid valve to record a His potential to identify the aorta using X-ray and/or CARTO visualization. Similarly, a decapolar catheter was placed in the coronary sinus (CS) serving as the timing and pacing reference [9].

### 2.4. Technique of TSP

The RF needle was chosen either as first option (*n* = 37 procedures) or after several failed attempts with a conventional Brockenbrough needle (*n* = 5 procedures). In challenging cases, transesophageal echocardiography (TOE) was also used to help the operator visualizing the correct position of the TSP. Single or double TSPs were performed and 1 or 2 long sheaths (either SL1- St. Jude Medical or TorFlex-Baylis Medical) were placed in the left atrium (LA) after demonstration of left atrial pressure by continuous invasive pressure measurement. All TSPs were done via the right femoral vein, except for one patient with complex congenital heart disease (#28/41 ASD, mitral valve replacement, repair, recurrent endocarditis, and finally metallic valve in atrial position and LAA in left ventricle continuity) and bilaterally blocked femoral access: in this case, the TSP was carried out from the right jugular vein.

### 2.5. RF TSP

The RF needle uses active radiofrequency applied to an insulated blunt-tipped electrode and has the same dimensions as a conventional Brockenbrough needle. Two side holes located 1.5 mm from the tip of the needle allow continuous intracardiac pressure reading. Using a dedicated RF generator (Baylis Medical), RF energy was applied using the default “pulsed” mode with monopolar RF power output for 300 milliseconds every second. If the LA could not be accessed using the “pulsed” mode, the operator could choose to apply RF energy in “continuous” mode for a duration of 2 seconds. A stackable 2 mm pin jumper cable is plugged into ports 1 and 2 on the pin block. The DuoMode Cable (Baylis Medical) is plugged into the stackable jumper cable in port 1. On the EAM system, the RF needle was defined as a 2Fr bipolar catheter, with 2 mm spacing centre to centre and 1 mm electrode width/length.

In some patients, the RF needle was advanced under fluoroscopic guidance to reach the fossa ovalis. When the operator was sure of the position, RF energy was applied according to the above indicated settings. If necessary, additional applications of RF energy were delivered to obtain LA access.

### 2.6. RF Needle Visualization on EAM

**Figure 1:**
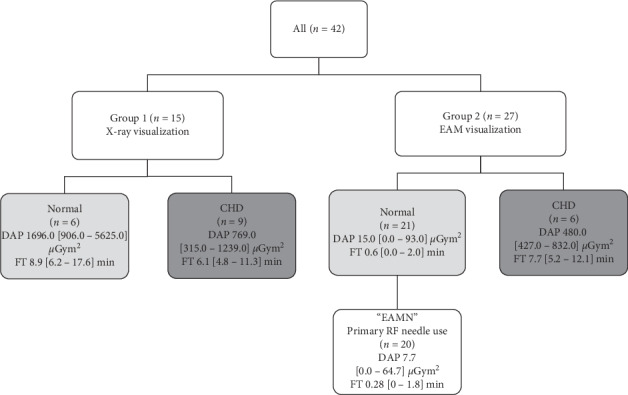
Flowchart of the different patient groups. DAP: Dose Area Product, FT: fluoroscopy time, EAM: electroanatomical mapping, and CHD: congenital heart disease.

**Figure 2:**
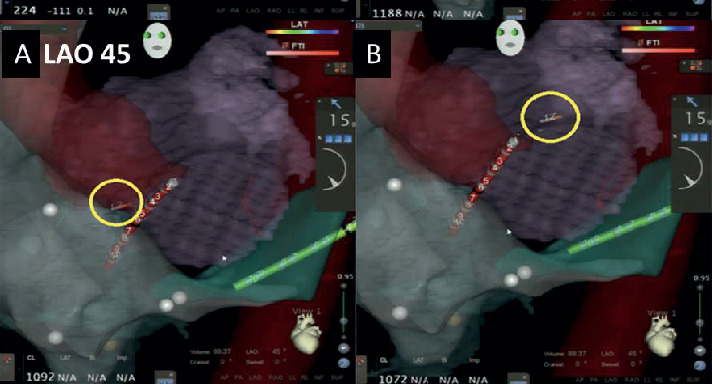
Direct visualization (yellow circle) of the needle tip on the electroanatomical mapping system (Left Anterior Oblique Projection): needle tenting on the fossa ovalis (A) and in the left atrium (B). FAM of right atrium (RA) merged with the 3D reconstruction of a preacquired CT scan and two decapolar catheters in His and coronary sinus (CS) position. Ao: aorta.

In a group of patients (Figure 1), a low fluoroscopy approach was used and the RF needle was visualized on the EAM system in order to allow 3D localisation of the RF needle tip. For this technique, a 3D map of the right atrium (RA) and CS was first acquired with fast anatomical mapping (FAM) using the mapping and ablation catheter (NaviStar ThermoCool, D/F or F/J SF ST, Biosense Webster, Brussels, Belgium) (Figure 2). This encoded the 3D space around the reconstructed chamber (the so-called matrix) and enabled localisation of noncensored catheters as well as the RF needle using the dedicated advanced catheter location (ACL) feature of the EAM system. Based on high-frequency low-power current emission from every electrode in each catheter connected to the EAM system and the six surface patches measuring these currents, the ACL calculates the position of each electrode with a mean accuracy of ∼3 mm [10]. The step-by-step approach has been previously described [11]. It is important to also create a FAM of the coronary sinus or alternatively of the left pulmonary artery in order to create enough matrix in the area of the LA. The use of preacquired 3D reconstructions is not necessary for any part of this visualization technique using the RF needle, but the mergence of the RA, LA, and aorta further facilitates the 3D orientation for the operator. Alternatively, the diagnostic catheter at the His region and inside the CS is equally well able to lineate the typical landmarks for the operator.

Given the wide heterogeneity of patients for anatomical conditions, we also selected a subgroup of patients with structural normal hearts and primary use of the RF needle visualized on the EAM (EAMN group).

### 2.7. Statistics

Continuous variables are presented as median and interquartile range (IQR). Categorical variables are expressed as absolute numbers and percentage. Continuous variables were compared using the Mann–Whitney test, while distribution of categorical variables was compared using the Chi square test. We used two-sided *p* value <0.05 to confirm statistical significance.

To demonstrate the learning curve for the operator using the RF needle for TSP, data from the entire cohort were used in an exponential regression analysis. In this regression analysis, the number of procedures is an ordinal variable, which was treated as continuous. A confirmatory comparison was made in EAMN group between the first 5 patients (early phase) and the last 5 (experienced phase).

All analyses were performed using R-package version 3.4.1 (http://cran.r-project.org/).

## 3. Results

### 3.1. Patients

From January 2015 to July 2017, an RF needle was used to gain left atrial access in a total of 41 consecutive patients (42 procedures) (Table 1; Figure 1) by a single operator. The included patients were predominantly males (60%) with a median age of 61 [47–69] years and a median body mass index of 27 [24–30]. Three patients had a mild renal dysfunction. Thirty-three patients presented with atrial fibrillation ((AF) 19 paroxysmal, 10 persistent, and 4 long standing persistent)), 8 with atrial tachycardia (AT), and one with ventricular tachycardia (VT). Twenty-three patients had structurally normal hearts, 14 had congenital heart disease (CHD), 3 had dilated cardiomyopathy, and 1 had significant valvular disease.  Table 2 lists the details of CHD conditions consisting of repaired atrial and ventricular septal defect (ASD and/or VSD, *n* = 9), tetralogy of Fallot (*n* = 1), congenitally corrected transposition of the great arteries (*n* = 1), cor triatriatum (*n* = 1), bicuspid aortic valve with coarctation of the aorta (*n* = 1), or operated congenital aortic stenosis (*n* = 1). CHD patients had significantly enlarged atrial chambers (median volume 132.5 [110–168] ml). Nine had previously undergone atrial septal closure (surgical or device).

More than half of the patients (22/41) had at least one previous TSP, known to cause fibrosis and stiffening of the interatrial septum [12].

Eventual success rate of TSP using the RF needle was 100%. When first RF needle TSP attempts were ineffective, the RF generator setting was changed from pulsed to continuous mode to help gaining LA access (3 procedures, 7%).

There were no immediate complications, specifically no postprocedural bleeding, relevant pericardial effusion, tamponade, oesophageal damage, or stroke.

### 3.2. Procedure Parameters

Median procedural time amounted to 147.5 [125–215] min with a median FT of 3.2 [0–7.4] min and median DAP of 199.5 [0–892.5] *µ*Gy.m [2]. The visualization of the RF needle on the EAM (*n* = 27 procedures, both CHD and structural normal heart patients) facilitated the procedure and allowed a low FT (median 0.88 [0–3.4] min) and DAP (median 33.5 [0–324.8] *µ*Gy.m [2]). Fluoroscopy exposure was even lower in the subgroup of patients with structural normal hearts and primary use of the RF needle visualized on EAM (group EAMN, *n* = 20; Figure 1) with a median FT of 0.28 [0–1.8] min and DAP of 7.7 [0–64.7] *µ*Gy.m [2].

### 3.3. Learning Curve for RF Needle TSP

**Figure 3:**
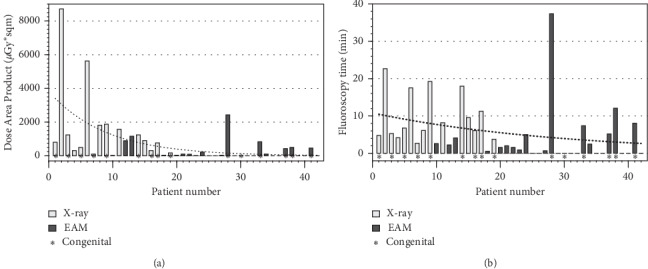
Case-by-case fluoroscopy time (FT) and Dose Area Product (DAP). Boxplots group 1 versus group 2: a significant reduction is evident for both DAP and FT in group 2 (needle visualized on EAM) compared to group 1 (standard fluoroscopy guidance of the needle). (a) Dose Area Product in consecutive patients. (b) Fluoroscopy time in consecutive patients.

Over the study period, the DAP significantly decreased in consecutive patients and there was a trend towards decrease of FT (Figure 3). All procedures were done by an expert operator using the same techniques. When analysing EAMN group, there was a significant decrease of both FT and DAP with a median FT of 2 [1.6–2.2] min for the first 5 cases versus 0.0 [0.0–0.0] for the last 5 cases (*P*=0.008) and a DAP of 36.8 [33.5–104.7] *µ*Gy.m [2] and 0.0 [0.0–0.0] for the last 5 cases (*P*=0.008). Procedure duration did not change over study period (131 [97–134] versus 135 [111–146] min for the first and last 5 patients, respectively, *P*=0.42).

## 4. Discussion

Our data confirms the application of RF needle in patients with various congenital or noncongenital cardiac conditions in achieving transseptal LA access. In addition, the visualization of the RF needle tip on the EAM system allows potentially lowering radiation exposure.

Although transseptal access can be achieved in most cases using a conventional Brockenbrough needle, we demonstrate herewith the use of the RF needle as an effective alternative, particularly for patients with CHD or multiple previous TSPs. About 20–30% of patients with structural normal hearts and paroxysmal AF ablation require at least one redo procedure, whilst patients with persistent and longstanding-persistent AF have even higher rates of reablation [13]. Repeated TSP is known to result in stiffening of the interatrial septum, making further TSP a technical challenge, frequently encountered in EP lab [12]. Children and adults with CHD present an even greater challenge to TSP mostly because of the presence of an artificial septum, due to either a surgical patch or device closure [14]. We found that the use of the RF needle serves as a valuable step-up in this situation, allowing for safe and feasible access. Given the growing number of CHD patients now reaching adulthood [15] and the higher percentage of redo ablations, this technique could play an increasingly important role. In our series, all TSPs employing an RF needle were successful, often following multiple failed attempts with a conventional needle. No complications occurred with the use of the needle.

### 4.1. 3D Visualization of the TSP

The 3D visualization of the needle tip on the EAM system added the benefit of a low fluoroscopy approach. The radiation time and exposure were very low (median FT under one minute) when the EAM visualization was exploited. In normal heart patients, using our low radiation approach with the needle visualization, the median FT decreased to 0.28 min. This approach appeared to be safe with no complications recorded. Moreover, eleven procedures were performed without using any radiation at all, relying completely on the EAM system. Finally, this approach is not expected by the authors to prolong the procedure since the RF needle provides more effective TSP, saving the time of multiple attempts, whereas the creation of the FAM of the RA requires only few minutes more for an experienced operator. Although we used preacquired 3D image reconstruction from CMR or CT in most of our cases, the visualization technique of the RF needle tip relies entirely on the creation of the 3D matrix when using the FAM feature, such that it can be performed independent of the availability of preprocedural imaging. Of note is that the LA geometry needs to be encoded in order to continuously see the RF needle icon inside the LA.

Due to the lack of immediate and manifest effects of radiation, fluoroscopy exposure seldom represents a major concern for the operator. Conversely, the help provided by fluoroscopic guidance becomes a strong rationale for its use, particularly in complex cases. However, the radiation injury hazard should not be underestimated, especially when considering the accumulating radiation exposure and the lifetime risk due to multiple procedures. The lifetime attributable excess cancer risk for patients undergoing repetitive procedures may be around 1 in 100^6^ and an association has been demonstrated between radiation related to cardiac procedures and incidence of malignancy also in the CHD population [16, 17]. The same risks seem to be attributable to the operators and recent reports have hinted at an excess risk of brain tumours among interventional cardiologists [6]. Remarkably, radiation concern seems to be a major motive, particularly for women, resulting in a preference for a career in noninterventional cardiology [18].

As shown by our experience, low fluoroscopy is possible and safe even in complex patients and it is facilitated by using additional tools to allow each part of the procedure to be carried out according to ALARA. Over the last decade, several studies have validated the capacity of EAM systems to decrease or even eliminate radiation exposure, also in left-sided procedures [19]. A further fluoroscopy reduction can be achieved by exploiting the 3D mapping system also for TSP, the main part still requiring X-ray. “Faking” the RF needle as a catheter using the ACL feature of the EAM system allowed the operator to localize the needle throughout the puncture in 3D with a very low or zero X-ray need. The RF needle (and in the same way any other noncensored catheter) can be visualized in real time after the FAM of RA is created. In addition, the FAM reconstruction of the distal CS encodes enough matrix to visualize the needle deep in the LA. Integrated CT or MRI imaging, although not essential, can facilitate the procedure as well, helping the operator to familiarize with the individual anatomy, particularly in congenital heart patients with complex anatomy.

### 4.2. Alternative Techniques

Alternative techniques to further decrease radiation use include the use of another imaging modality that is echocardiography, such as intracardiac echo (ICE) or 3D transoesophageal echocardiography (TOE) [20, 21]. Both techniques permit a direct view of the atrial septum and thus the localisation of a safe site for the puncture: this represents an added value, especially in CHD patients where the modified anatomy complicates the usual anatomical landmarks and manoeuvres. However, the adoption of all these approaches has been poor, probably due to issues regarding complexity and the additional time added to already lengthy procedures. Also, in many laboratories worldwide and especially in Europe, ICE or 3D TOE (plus dedicated echo operator) may not be available due to excessive costs.

### 4.3. Learning Curve

Abandoning the “safe waters” of the standard approach is not easy and it requires dedication and acceptance of a learning curve, even for very experienced operators in fluoroscopy-based electrophysiology. Our approach has developed over time while accumulating experience in needle manipulation and its visualization on EAM. With the growing experience, fluoroscopy time decreased gradually to the point where it was possible to perform some ablations without any X-ray at all and using EAM guidance only. Once the operator feels comfortable that the fluoroscopy image just depicts the same as the real-time EAM visualization, the need for confirmation using fluoroscopy gets smaller and smaller as we have shown in this study. As a safety feature, the operator can of course always decide to have a quick glance. Most likely, there is no difference in risk from very low to zero radiation exposure for the patient. And of course, the safety of the procedure needs to be ascertained at all times and the achievement of reduced radiation exposure should result in an increase of risk.

### 4.4. Limitations

This is an observational retrospective study including a small number of patients. Nevertheless, our work includes patients with a large variety of cardiac conditions and analyses a new technique employing the needle visualization on the EAM. The low/zero fluoroscopy approach described herewith appears to be safe but will need validation and refinement in a prospective multicentre and multioperator trial. Finally, the retrospective design did not allow us to collect the time to achieve LA access and evaluate potential lengthening/shortening of the procedure using this new approach versus the conventional one.

## 5. Conclusion

RF needle constitutes an effective alternative to the conventional Brockenbrough needle when transseptal access of the LA is required, also in challenging patients with multiple previous TSP or artificial patches. Moreover, the needle tip visualization in combination with the EAM allows a low or even zero fluoroscopy approach.

## Figures and Tables

**Table 1:** Patient demographics overall.

	Median age (IQR)	Gender	Median BMI (IQR)	Arrhythmia type	Additional information
Structurally normal heart *N* = 23 pts	64 (52–69)	10F/13M	27.9 (24.5–31.1)	AF = 21 pts AT = 2 pts	12 pts with at least one previous TSP

Dilated cardiomyopathy *N* = 3 pts	52 (44–63)	3M	26.3 (24.0–27.4)	VT = 1 pt AF = 2 pts	1 pt with previous TSP

Valvular heart disease *N* = 1 pt	36	M	30.9	AF	Marfan syndrome, aortic root replacement + resuspension of aortic valve, MV and TV repair, and previous AF ablation

Adult congenital heart disease *N* = 14 pts	58 (46–66)	7F/8M	25.5 (23.2–27.5)	AF = 8 pts AT = 6 pts	9 pts with artificial septal closure (surgical or device); 9 pts with at least one previous TSP

IQR: interquartile range, BMI: body mass index, M: male, F: female, VT: ventricular tachycardia, AT: atrial tachycardia, AF: atrial fibrillation, and MV: mitral valve.

**Table 2:** Cases description for CHD conditions.

Proc	Arrhythmia type	LA volume (ml)	TSP route (femoral/jugular)	Anatomy	Cardiac history and previous interventions
1	Paroxysmal AF	81	Femoral	AVSD	Partial AVSD + secundum ASD repair: VSD and ASD (fenestrated Dacron patch) closure + bidirectional Glenn. Hypoplastic RV

3	Paroxysmal AF	135	Femoral	ASD + mitral	Mitral valvuloplasty, ASD closure with Amplatzer and previous AF ablation

5	Paroxysmal AF	90	Femoral	ASD	Secundum ASD surgical repair, PM implanted, and previous AF ablation

7	AT	130	Femoral	ASD	ASD repair; PM implanted

9	AT	95	Femoral	VSD, coarctation, and bicuspid Ao	Bicuspid aortic valve AVR Mech + root, subaortic stenosis coarctation aorta repaired with redo, residual VSD, PM implanted, and 2 previous AT ablations

14	Persistent AF	190	Femoral	AVSD + mitral	Common atrium with AVSD: repair of atrioventricular septal defect with Dacron patch, left atrioventricular valve repair, MV replacement with 27 mm bileaflet mechanical valve, and more than 60 DCCV in life

16	AT	110	Femoral	CCTGA	CHB 29 mm St Jude TVR, VSD patch, PFO closure, LA cryoablation, CRT-D implanted, and previous AT ablation

17	Persistent AF	150	Femoral	ASD	Secundum ASD surgical closed and previous AF ablation

19	Persistent AF	127	Femoral	Cor triatriatum	No surgical intervention

28 + 41	AT	120	Jugular	ASD + mitral	ASD and MV repair (parachute MV), recurrent endocarditis and finally metallic valve in atrial position and LAA in LV continuity, and bilaterally blocked femoral veins

30	Paroxysmal AF	152	Femoral	AS	Congenital aortic stenosis: valvectomy aorta and myomectomy, aortic valve and ascending replacement, and previous AF ablation

33	AT	258	Femoral	ASD	Secundum ASD closed and 3 previous AT/AF ablations

37	Persistent AF	168	Femoral	TOF	Tetralogy Fallot repaired, previous AF and AFL ablations, and CRT-D implanted (PM dependent)

38	Persistent AF	209	Femoral	VSD	VSD closure with Amplatzer and 3 previous AF ablations

LA: left atrium, AVSD: atrioventricular septal defect, ASD: atrial septal defect, VSD: ventricular septal defect, RV: right ventricle, PM: pacemaker, AVR: aortic valve replacement, Mech: mechanical, Ao: aorta, CCTGA: congenitally corrected transposition of the great arteries, CHB: complete heart block, PFO: persistent foramen ovale, TVR: tricuspid valve replacement, TOF: tetralogy of Fallot, and LAA: left atrial appendage.

## Data Availability

Data are available from the corresponding author upon request.
